# Expanding Usutu virus circulation in Italy: detection in the Lazio region, central Italy, 2017 to 2018

**DOI:** 10.2807/1560-7917.ES.2019.24.3.1800649

**Published:** 2019-01-17

**Authors:** Fabrizio Carletti, Francesca Colavita, Francesca Rovida, Elena Percivalle, Fausto Baldanti, Ida Ricci, Claudio De Liberato, Francesca Rosone, Francesco Messina, Eleonora Lalle, Licia Bordi, Francesco Vairo, Maria Rosaria Capobianchi, Giuseppe Ippolito, Giuseppina Cappiello, Alberto Spanò, Silvia Meschi, Concetta Castilletti

**Affiliations:** 1 Laboratory of Virology, National Institute for Infectious Diseases ‘Lazzaro Spallanzani’ IRCCS, Rome, Italy; 2 Molecular Virology Unit, Microbiology and Virology Department, Fondazione IRCCS Policlinico San Matteo, Pavia, Italy; 3 Department of Clinical, Surgical, Diagnostic and Pediatric Sciences, University of Pavia, Italy; 4 Istituto Zooprofilattico Sperimentale delle regioni Lazio e Toscana, Rome, Italy; 5 Regional Service for Surveillance and Control of Infectious Diseases (SERESMI)-Lazio Region, National Institute for Infectious Diseases ‘Lazzaro Spallanzani’ IRCCS, Rome, Italy; 6 Scientific Direction, National Institute for Infectious Diseases ‘Lazzaro Spallanzani’ IRCCS, Rome, Italy; 7 Unit of Microbiology, Sandro Pertini Hospital, Rome, Italy

**Keywords:** Usutu virus, flavivirus, blood donation screening, emerging viruses, arbovirus, Italy, blood-borne infections, vector-borne infections, zoonotic infections, viral infections, Flaviviridae, West Nile virus, infection control, surveillance, laboratory

## Abstract

Blood donation screening for West Nile virus (WNV) was mandatory in the Lazio region in 2017 and 2018 (June-November) according to the national surveillance plan. In these years, all five donations reactive in WNV nucleic acid amplification tests harboured instead Usutu virus (USUV). Clade ‘Europe 2’ was identified in four blood donations and a 2018 mosquito pool. The cocirculation of WNV and USUV in Lazio warrants increased laboratory support and awareness of possible virus misidentification.

In the summer and autumn seasons of 2017 and 2018, following serological identification of West Nile virus (WNV) infection in horses, mandatory WNV screening with nucleic acid amplification test (NAT) was established for blood donations in some provinces of the Lazio Region in central Italy: Viterbo (starting from 18 August 2017), Latina (12 September 2018) and Rome (10 October 2018). During this period, five blood donations were reactive in WNV NAT screening. In none of these samples could WNV positivity be established by further tests conducted at the Regional Reference Laboratory. 

In the present study, we describe serological, detailed molecular and phylogenetic analyses undertaken to better characterise the WNV positivity observed during the screening. 

## Processing of blood samples

For the Lazio Region, centralised blood screening for WNV is performed at the Biological Qualification Center (CQB) for NAT blood screening at the Sandro Pertini Hospital in Rome, using NAT (Cobas WNV NAT screening test, Roche, Mannheim, Germany). Blood donations reactive in WNV NAT screening and confirmed twice by the same method at the CQB, are considered not suitable for the transfusion service and are discarded. Plasma aliquots of these donations are sent to the Regional Reference Laboratory for Arbovirosis (Laboratory of Virology, National Institute for Infectious Diseases L. Spallanzani, Rome) for confirmatory testing and further diagnostic investigation. Donors who provided WNV NAT-positive donations are recalled 2–3 weeks after donation, to complete serological investigation.

All five donors reactive in repeated WNV NAT were asymptomatic and fulfilled the eligibility criteria concerning WNV risk. According to Ministerial Decree of 2 November 2015, in order to guarantee self-sufficiency in blood components during the summer period, it is recommended to use the WNV NAT test as an alternative to the provision of 28 days suspension of donors who have spent at least one night in areas at risk of WNV infection [1]. The areas with a documented WNV circulation are established by an appropriate epidemiological surveillance system. The main demographical data of these donors are shown in the Table (Donors #1 to #5). [Table t1]

**Table t1:** Details of Usutu virus infection in blood donors, Lazio and Lombardy regions, Italy, 2017–2018 (n = 9)

Donor^a^	Age group (years)	Year/month of donation	Travel history in previous month	Place of residence	Cobas WNV assay (Ct)	WNV serology^b^ (titre^c^) IgG / IgM		USUV serology^d^ (titre^c^) IgG / IgM	
						T0	FU	T0	FU
#1	30–39	2017/8	Switzerland	Argentina	36.3	1:160	1:160	1:160	1:640
						1:40	1:40	<1:20	1:40
#2	40–49	2017/9	No travel	Viterbo (Lazio, Italy)	37.3	<1:20	1:40	<1:20	1:640
						<1:20	1:40	<1:20	1:160
#3	20–29	2018/8	Sardinia (Italy)	Frosinone (Lazio, Italy)	33.5	<1:20	<1:20	<1:20	<1:20
						ND	ND	ND	ND
#4	60–69	2018/9	No travel	Cisterna di Latina (Lazio, Italy)	33.4	<1:20	<1:20	<1:20	<1:20
						ND	ND	ND	ND
#5	40–49	2018/9	No travel	Cisterna di Latina (Lazio, Italy)	37.9	<1:20	<1:20	<1:20	<1:20
						<1:20	≤1:20	<1:20	1:40
#6	60–69	2017/8	No travel	Cremona (Lombardy, Italy)	NA	Neg	Pos	<1:10	1:160
						Neg	Pos	< 1:10	1:20
#7	50–59	2017/8	Emilia Romagna, Marche (Italy)	Milano (Lombardy, Italy)	NA	Neg	Pos	<1:10	1:160
						Pos	Pos	1:40	1:10
#8	30–39	2017/8	Veneto (Italy)	Cremona (Lombardy, Italy)	NA	Neg	Pos	<1:10	1:160
						Neg	Pos	<1:10	1:20
#9	30–39	2017/8	Lazio (Italy)	Milano (Lombardy, Italy)	NA	Neg	Pos	<1:10	1:160
						Neg	Pos	<1:10	1:10

Ct: cycle threshold; FU: at follow-up (2–3 weeks after donation); NA: not available; ND: not done; Neg: negative; Pos: positive; T0: at time of blood donation; USUV: Usutu virus; WNV: West Nile virus.

^a ^One donor from Lazio was female, the other eight donors were men.

^b^ Serological investigation for WNV was performed with commercially available kits: indirect immunofluorescence assay (anti-West Nile virus IFA IgM and IgG, Euroimmun, Germany) for patients #1–#5 and ELISA (West Nile Virus IgM/IgG Capture DxSelect Focus Diagnostics, Diasorin, United States) for patients #6–#9.

^c ^The titres are expressed as highest serum dilution showing specific immunofluorescent staining. For WNV ELISA, the test results are expressed as pos/neg.

^d ^Anti-USUV IgM and IgG antibodies were determined by immunofluorescence assay using home-made glass slides, prepared with a mix of uninfected and USUV-infected Vero E6 cells, according to standard procedures established for flaviviruses [20].

## Virological characterisation of WNV NAT-reactive samples

Plasma samples were concentrated by ultracentrifugation and tested with a nested pan-flavivirus-RT-PCR targeting the NS5 gene (modified from [2], amplicon size: 210 nt), followed by amplicon sequencing. New sequences described in this report have been registered in GenBank under accession numbers MK015649, MK024375, MK006173, MK006174, MK006175, MK060108, MK060109, MK060110 and MK060111.

**Figure 1:**
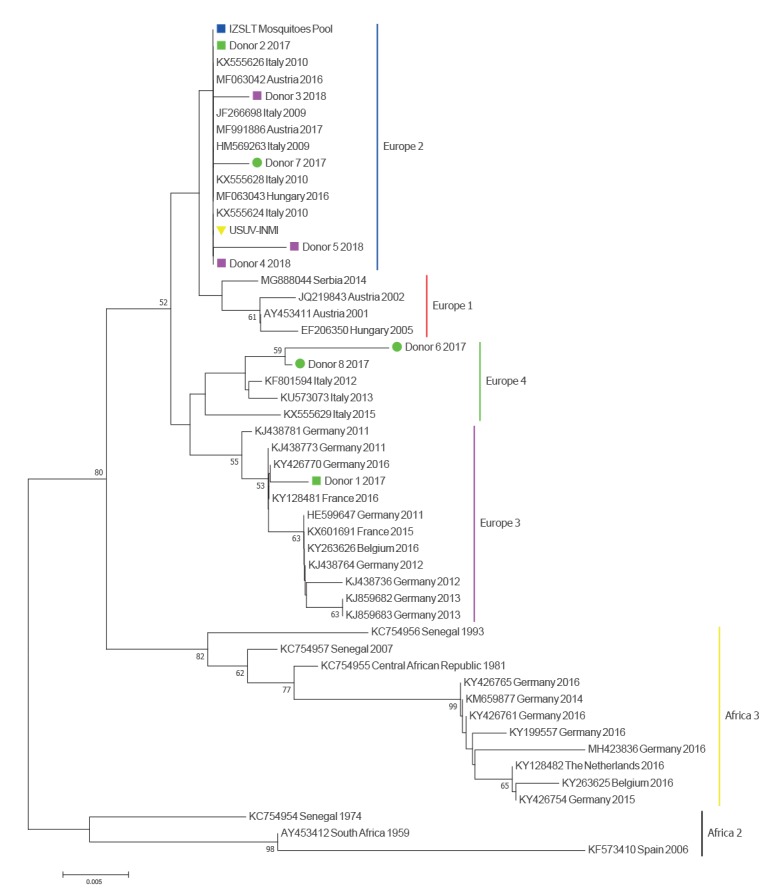
Phylogenetic analysis of Usutu virus strains, neighbour-joining method, central and northern Italy, 2017–2018 (n = 50)

**Figure 2:**
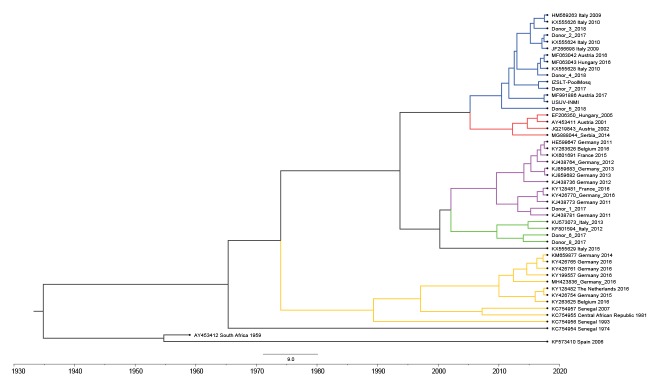
Phylogenetic analysis of Usutu virus strains, Bayesian maximum clade credibility, central and northern Italy, 2017–2018 (n = 50)

The sequencing results indicated that all five blood donations harboured Usutu virus (USUV) and not WNV. These results were further confirmed with a USUV-specific real-time RT-PCR, modified from Nikolay et al. [3], and by an USUV-specific nested RT-PCR targeting NS5, followed by sequencing. The phylogenetic tree, built with both neighbour-joining and Bayesian maximum clade credibility approaches, indicated that the USUV strains detected in four of the five blood donors from the Lazio region belonged to the clade ‘Europe 2’, while the strain detected in donor #1 belonged to the clade ‘Europe 3’ (Figure 1 and 2).

Phylogenetic tree based on the partial NS5 gene (position 9004–9406 of HM569263), built using the neighbour-joining method. The evolutionary distances were computed using the Kimura 2-parameter method. The rate variation among sites was modelled with a gamma distribution (shape parameter = 0.25). Each record consists of accession number, place and year of detection/isolation. Phylogenetic analysis was conducted in the MEGA7 software package.

Green squares: donors from Lazio in 2017; green circles: donors from Lombardy in 2017; pink squares: donors from Lazio in 2018; blue square: mosquito pool collected in Lazio in 2018; yellow triangle: Usutu virus-positive control available at the National for Infectious Disease L. Spallanzani.

Colour code of Usutu virus clades: red: Europe 1; blue: Europe 2; pink: Europe 3; green: Europe 4; yellow: Africa 3; black: Africa 2.

Bayesian maximum clade credibility tree, obtained using the Bayesian Evolutionary Analyses Sampling Trees (BEAST v 1.7.5) software [19], calibrated with the most ancient strain AY453412 South Africa 1959. It was run using 100 × 10^6^ generations with Hasegawa–Kishino–Yano + three codon positions substitution model, the gamma distribution for the rate variation among sites, the strict clock model and constant size tree model. In this tree, all multifurcations of the neighbour-joining tree were resolved into sequential bifurcations.

Colour code of Usutu virus clades: red: Europe 1; blue: Europe 2; pink: Europe 3; green: Europe 4; yellow: Africa 3; black: Africa 2.

The serological investigation revealed that four donors did not have WNV- or USUV-specific antibodies at donation, while donor #1 had both IgG and IgM specific for WNV. In follow-up samples, obtained 2–3 weeks apart, USUV seroconversion was observed for donors #2 and #5, while the USUV-specific antibody titre exceeded the WNV-specific antibody titre in donor #1 (Table).

## Vector investigation

According to the national integrated surveillance plan for WNV and USUV, the Istituto Zooprofilattico Sperimentale delle Regioni Lazio e Toscana performed the entomological investigation: adult mosquitoes were sampled at sites with known virus circulation (areas where horses positive for anti-WNV antibodies or blood donors reactive in WNV NAT had been identified) using traps of the model Italian Mosquito Trap (IMT; PeP, San Giuliano Milanese, Italy), BG Sentinel (BioGents, Regensburg, Germany) and Gravid (BioQuip Products, Rancho Dominguez, United States). The sampling protocol was defined depending on the number of WNV cases in the area and on the number of caught mosquitoes. After mosquito sorting and identification, those of the species *C. pipiens* were divided in pools of at most 100 specimens and analysed by RT-PCR for virus detection. 

In the period between September and October 2018, 47 trapping exercises were performed at nine sites with known virus circulation in the provinces of Rome, Latina and Frosinone. A total of 2,443 specimens of *C. pipiens* were caught, divided into 38 pools and tested by RT-PCR for the presence of WNV and USUV RNA. Among the 38 tested pools, 14 were positive for USUV (minimum infectious rate: 1.3); they originated from two municipalities in the Latina province. Sequencing indicated that these viruses belonged to the ‘Europe 2’ clade and were very similar to those obtained from blood donors #2 to #5 (Figure 1).

During the same period, three horses with neurological signs and positive WNV serology were located in the province of Latina. One horse died, and WNV lineage 2 was detected by RT-PCR in its brain (cerebellum and medulla oblongata). These results were confirmed by the National Reference Centre for Foreign Animal Diseases (CESME), Istituto Zooprofilattico Sperimentale dell’Abruzzo e del Molise.

## Characterisation of Usutu virus strains detected in blood donors from northern Italy

We also performed molecular analysis on four USUV-positive blood donations identified in 2017 by the Regional Reference Laboratory of the Lombardy Region (Molecular Virology Unit, Fondazione IRCCS Policlinico San Matteo Pavia) in northern Italy, where endemic cocirculation of different USUV strains was reported [4]. Their main demographical data are shown in the Table. We identified the ‘Europe 2’ clade in one donor (donor #7) and the ‘Europe 4’ clade in two donors (donors #6 and #8) (Figures 1 and 2). For the fourth donor, only the short pan-flavivirus RT-PCR amplicon could be sequenced; this sequence clustered with the ‘Europe 2’ clade.

## Discussion

USUV is a mosquito-borne flavivirus, member of the Japanese encephalitis virus group, with six distinct clades so far recognised [5]. Its natural life cycle involves mosquitos as vectors and birds as amplifying hosts. Humans and horses are dead-end hosts, as for WNV.

USUV was isolated in South Africa in 1959 and was first reported in Europe in 2001, causing mass mortality of Eurasian blackbird (*Turdus merula*); however, a retrospective study showed that it has been present in Italy at least since 1996 [6]. Subsequently, USUV spread to other country, including Hungary (2003–2006), Switzerland (2006), Spain (2003–2006), Germany (2010), Czech Republic (2014), Belgium, France and the Netherlands (2016), where the virus was detected or isolated from mosquitos, birds and bats [7-10].

The first human USUV infection was reported in the Central African Republic in 1981 [11]; the first neuro-invasive human infection in Europe was reported in Italy in an immunocompromised host in 2009 [12]; several cases of disease in humans were described thereafter [13-15].

Recently, USUV circulation has been increasing in several Europe regions, overlapping with areas endemic for WNV that exploits the same main mosquito vector [16]. Molecular and serological evidence of USUV circulation has been reported in northern Italy, involving the ‘Europe 2’ and the ‘Europe 4’ clades [4] and infecting birds, mosquitos and humans; human infections have been both asymptomatic and symptomatic [13].

An annual surveillance plan is issued by the Italian Ministry of Health with the aim of monitoring WNV and USUV circulation and triggering appropriate public health measures. The purpose is the limitation of virus spread, control of the vector population and elimination of transmission through blood, blood components, tissues and organ donation during the period of increased vector activity in the summer and autumn season (June–November) [1]. In 2017 and 2018, respectively two and three blood donations from the Lazio region were positive in WNV NAT screening. Molecular characterisation revealed that the WNV NAT positivity was indeed due to the presence of USUV RNA. Intensified entomological surveillance for arboviruses detected 14 mosquito pools that were positive for USUV RNA.

Partial molecular characterisation indicated close genetic relationships between human and mosquito USUV strains detected in Lazio region; all belonged to the ‘Europe 2’ clade. The only USUV strain of clade ‘Europe 3’, detected in donor #1, was presumably acquired in Switzerland, according to the travel history of the donor; in addition, this donor’s place of residence, Argentina, accounted for the serological pattern observed at donation, that was consistent with previous exposure to other related flaviviruses.

The presence of WNV lineage 2 detected by molecular assay in the brain of a symptomatic horse in the province of Latina is evidence of the cocirculation of WNV and USUV in this area. Data from blood donors in Lombardy confirmed the cocirculation, in northern Italy, of different USUV strains (belonging to clades ‘Europe 2’ and ‘Europe 4’). This finding is in line with previous reports [13] and indicates autochtonous circulation after multiple importation events.

This is the first report showing circulation of USUV virus in the Lazio region, both in mosquito vectors and in human hosts, while so far, USUV has not been detected in other animal hosts (neither equids nor birds).

This study corroborates the hypothesis that USUV can cause clinically asymptomatic infection in humans [17]; this, in turn, may be of concern regarding the safety of the blood supply in areas where the virus is active. Although there is no evidence of human-to-human transmission of USUV in the transfusion (or transplant) setting, asymptomatic infected donors may donate USUV-infectious blood, which may cause severe disease in immunocompromised patients [18].

Public health authorities, blood transfusion services and clinicians should be aware of the expanding risk of USUV infection in humans, especially during the summer, and advanced laboratory support is required to address the problem of possible virus misidentification by screening tests.
